# Analysis of Specialty Nephrology Care Among Patients With Chronic Kidney Disease and High Risk of Disease Progression

**DOI:** 10.1001/jamanetworkopen.2022.25797

**Published:** 2022-08-19

**Authors:** Maggie Wang, Samson S. Peter, Chi D. Chu, Delphine S. Tuot, Jonathan H. Chen

**Affiliations:** 1 Department of Biomedical Data Science, School of Medicine, Stanford University, Stanford, California; 2 School of Medicine, Stanford University, Stanford, California; 3 Department of Medicine, University of California, San Francisco; 4 Division of Nephrology, University of California, San Francisco at Priscilla Chan and Mark Zuckerberg San Francisco General Hospital, San Francisco; 5 Center for Innovation in Access and Quality, University of California, San Francisco; 6 Stanford Center for Biomedical Informatics Research and Division of Hospital Medicine, Department of Medicine, Stanford University, Stanford, California

## Abstract

**Importance:**

Identification of patients with chronic kidney disease (CKD) with high risk of progression to kidney failure can help ensure they receive appropriate and effective nephrology care.

**Objective:**

To examine whether patients with CKD at various levels of kidney failure risk receive nephrology care within 1 year of established risk.

**Design, Setting, and Participants:**

This population-based, retrospective cohort study collected nationwide administrative health claims data from 156 733 adult patients who met the Kidney Disease: Improving Global Outcomes initiative CKD diagnostic criteria between January 1, 2012, and December 31, 2019, and had an available urine albumin to creatinine ratio within 90 days of a serum creatinine laboratory test. Patients with a history of dialysis or kidney transplant, a prior visit with a nephrologist in the past year, or palliative care billing codes or those who died or disenrolled within 1 year of the albumin to creatinine ratio measurement were excluded. Data analysis was performed from September 10, 2022, to February 14, 2022.

**Exposures:**

Kidney failure risk computed with the 5-year Kidney Failure Risk Equation.

**Main Outcomes and Measures:**

The main outcome was nephrology care rates across tiers of kidney failure risk, estimated as the proportion of individuals having a nephrologist visit within 1 year after index time.

**Results:**

The study population consisted of 156 733 patients with CKD (mean [SD] age, 74.6 [8.4] years; 91 906 [58.6%] female; 86 457 [55.2%] White). A total of 106 004 patients (67.6%) had a low (≤1%) 5-year risk of kidney failure. Nephrology visit rates increased with higher kidney failure risk. Among the 137 highest-risk patients, 79 (57.7%; 95% CI, 48.4%-64.7%) had a nephrology visit. Among 7730 patients with risk above a 10% threshold, 3208 (41.5%; 95% CI, 40.3%-42.4%) had a nephrology visit.

**Conclusions and Relevance:**

This study’s findings suggest that nearly half of patients with CKD at high risk of progressing to kidney failure do not have a nephrologist visit within 1 year of established risk. These findings have implications in the design of risk-based guidelines for referral and in the practice of delivering nephrology care to patients with CKD.

## Key Points

**Question:**

Are patients with chronic kidney disease who are at high risk of kidney failure receiving nephrology care within 1 year of established risk?

**Findings:**

In this cohort study of 156 733 adult patients with chronic kidney disease, 58% of patients at high risk of progressing to kidney failure had a nephrology visit within 1 year of established risk.

**Meaning:**

These findings suggest that many patients with chronic kidney disease at high risk of kidney failure do not receive nephrology care within 1 year of established risk; better strategies are needed for identifying and referring high-risk patients.

## Teaser

This cohort study examines rates of nephrology care among patients with chronic kidney disease with different levels of risk of kidney failure.

## Introduction

An estimated 37 million adults in the US have chronic kidney disease (CKD).^1,2^ Major complications of CKD include progression to kidney failure and increased risk of cardiovascular disease (CVD), which ultimately contribute to substantial morbidity and mortality.^3,4,5,6^ Early referral to a nephrologist for disease management is associated with reduced burden and severity of comorbid diseases, fewer hospitalizations, and greater length of survival.^7,8^ Despite awareness surrounding the benefits of referring high-risk patients, deficiencies in risk identification and patient referral persist.^9^ High-risk patients who do not receive appropriate nephrologist attention represent a missed opportunity for the delivery of interventions to reduce CKD progression.

The Kidney Disease: Improving Global Outcomes (KDIGO) guidelines and several other clinical guidelines^10,11^ recommend nephrology referrals based on laboratory tests, including serum creatinine and urine albumin to creatinine ratio (ACR). However, strict adherence to the KDIGO referral recommendations may be infeasible when they would likely result in referrals far outnumbering the supply of nephrology care at a tertiary care center.^12^ A supply-demand mismatch in even a well-resourced medical center suggests that the KDIGO guidelines may be impractical in lower-resourced institutions as well.

Risk estimation tools that target referral care for high-risk patients can potentially address the shortcomings of guidelines that rely on laboratory-based criteria. The Kidney Failure Risk Equation (KFRE) estimates a patient’s risk of progression to kidney failure and has been externally validated across multinational populations.^13,14^ Using a patient’s age, sex, estimated glomerular filtration rate (eGFR), and ACR, the KFRE outputs a risk score that estimates the likelihood of progressing to kidney failure within the next 2 or 5 years.^13^ A study^15^ in Manitoba, Canada reported shortened wait times for nephrology referrals by triaging referral requests with a 3% 5-year KFRE risk cutoff and redirecting patients with risk below the cutoff to primary care management. Studies^16,17^ in the UK and the US Veterans Affairs system have shown that referrals based on the KFRE risk compared with traditional criteria could result in substantial reallocation of which patients are being referred. Another UK study^10,18^ found that combining a 5% 5-year KFRE risk cutoff with the National Institute for Health and Care Excellence referral guidelines would more effectively identify patients at high risk of progression to kidney failure while minimally altering referral volumes.

Our objective was to examine the gap in nephrology care between patients with CKD at predictably high risk for kidney failure and those receiving treatment from a nephrologist. We analyzed rates of nephrology visits across KFRE risk levels in a large US national cohort, focusing on patients not yet receiving nephrology care at the time when their risk became identifiable by the KFRE.

## Methods

We performed a retrospective cohort study using the Optum Clinformatics Data Mart (Optum CDM), version 8.1, a database derived from a large adjudicated claims data warehouse (Optum Inc). Optum CDM consists of nationwide administrative health claims for approximately 15 million to 20 million individuals covered by a commercial health plan and members of Medicare Advantage between January 1, 2007, and March 31, 2021. This study was approved by the Stanford University Institutional Review Board. As a retrospective study on administrative data produced for nonresearch purposes, the Stanford University Institutional Review Board has granted a waiver of consent, a waiver of assent, and a waiver of Health Insurance Portability and Accountability Act authorization. This study followed the Strengthening the Reporting of Observational Studies in Epidemiology (STROBE) reporting guideline.

### Study Population

The KDIGO guidelines define CKD as prolonged abnormal kidney function, which may manifest as 2 eGFR values of less than 60 mL/min/1.73 m^2^ taken at least 90 days apart.^11^ We identified all patients older than 18 years who met this diagnostic criterion between January 1, 2012, and December 31, 2019. Participant race and ethnicity were determined from the Optum database’s combined race and ethnicity variable. Race and ethnicity were used in this study to perform subgroup analysis. We calculated eGFR with the 2021 Chronic Kidney Disease Epidemiology Collaboration equation, which uses age, sex, and serum creatinine level as inputs.^19^

We then identified patients with at least 1 urine ACR laboratory result measured within ±90 days of an available second eGFR value. Patients with an ACR and eGFR pair of results had a calculable KFRE. We allowed for a flexible 90-day window between ACR and eGFR because laboratory tests are often not completed simultaneously.

Index time was defined as the date of the patient’s earliest calculable KFRE. If the eGFR and ACR were not measured on the same date, then the later date was used as the index time. We excluded patients who had already progressed to kidney failure at index time, defined as those receiving dialysis treatment or kidney transplant or those with an eGFR less than 15 mL/min/1.73 m^2^. Dialysis and kidney transplant procedures were extracted using *International Classification of Diseases, Ninth Revision* (*ICD-9*) and *International Classification of Diseases and Related Health Problems, Tenth Revision, Procedure Coding System* (*ICD-10-PCS*) procedure codes. To evaluate incident nephrology care, our primary analysis excluded patients who had already received nephrology care within 1 year before index time. In addition, we excluded patients who had any recorded palliative care *ICD-9* or *ICD-10-PCS* diagnosis codes, because these patients may have had appropriate reasons not to pursue nephrology care. Finally, our primary analysis excluded patients who died or disenrolled from their health care plan within 1 year of index time.

### Study Design

Our goal was to determine how often patients in our cohort received nephrologist care within 1 year of possible kidney failure risk stratification. Although the CKD diagnosis might not have been incident at the time of risk stratification, high-risk patients can benefit from incident nephrology care even if their diagnosis is not new. We computed KFRE risk scores for patients using published coefficients from the KFRE model for 5-year risk of kidney failure.^13,15,20^ To ensure that KFRE risk served as a credible estimate of true kidney failure risk, we plotted and visually evaluated calibration curves of observed vs estimated risk. Patients were subdivided into risk tiers of 10% according to their KFRE risk. Most patients fell in the 0% to 10% risk range, so we further categorized these patients into narrower risk groups of 0% to 1%, greater than 1% to 2%, greater than 2% to 3%, greater than 3% to 5%, and greater than 5% to 10%. Within each risk group, we estimated the nephrology visit rate as the proportion of patients with a visit to a nephrologist in the 1-year period after their index time. We repeated our analysis in subgroups by race and ethnicity (Black, Hispanic, and White, excluding patients of Asian and unknown race and ethnicity because of small patient numbers), sex (male or female), index year (2012-2019, in 2-year increments), and age (<70, 70-79, or ≥80 years).

To evaluate the potential impact of using a KFRE risk threshold for referral, we estimated visit rates relative to a set of risk thresholds. Previous studies^15,17^ have proposed using risk thresholds of 3% and 5%. We analyzed both these suggested thresholds and included an additional “relaxed” 10% threshold to examine whether a less aggressive threshold for referral might still alter visit rates. For each threshold, we computed the nephrology visit rate in patients with risk at or below the threshold.

### Statistical Analysis

In a sensitivity analysis, we excluded patients with a nephrology visit within 3 years before index time, extending the 1-year exclusion time frame. To analyze prevalent instead of incident nephrology care, we repeated our primary analysis without excluding patients with a nephrology visit in the year prior. We performed a Kaplan-Meier analysis to determine the cumulative incidence of nephrology visits, which allowed us to include patients who were censored because of death or disenrollment. Because many patients lacked ACR testing, we also conducted a sensitivity analysis in which we included patients who had a urine protein-to-creatinine ratio measurement, which we converted to ACR using a validated conversion equation.^21^ To further explore how missing ACR may have affected the results of our primary analysis, we analyzed nephrology visit rates in patients for whom ACR was absent and stratified based on eGFR ranges. Finally, we repeated our primary analysis using the 2009 Chronic Kidney Disease Epidemiology Collaboration equation for eGFR, which was used when KFRE was originally developed but is now discouraged because of its inclusion of a Black/non-Black race variable.^19,22^ Detailed methods for sensitivity analyses are available in the eMethods in the Supplement.

Data analysis was performed from September 10, 2022, to February 14, 2022. All analyses were performed in Python software, version 3.8 (Python Software Foundation).

## Results

### Primary Analysis

**Figure 1.**
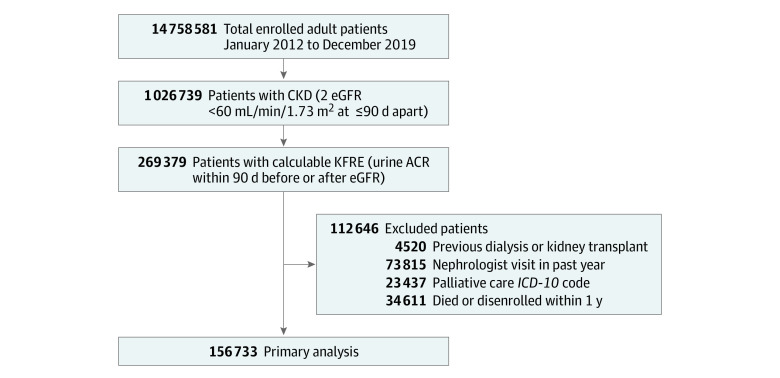
Flow Diagram for Cohort Selection ACR indicates albumin to creatinine ratio; CKD, chronic kidney disease; eGFR, estimated glomerular filtration rate; *ICD-10*, *International Classification of Diseases and Related Health Problems, Tenth Revision, Procedure Coding System*; and KFRE, Kidney Failure Risk Equation.

In total, we identified 156 733 unique adult patients (mean [SD] age, 74.6 [8.4] years; 91 906 [58.6%] women and 64 827 [41.4%] men; 7281 [4.6%] Asian, 24 891 [15.9%] Black, 29 658 [18.9%] Hispanic, 86 457 [55.2%] White, and 8446 [5.4%] of unknown race or ethnicity) with CKD and calculable KFRE who had at least 1 year of follow-up and who did not meet the exclusion criteria (Table 1). Derivation of the study population is shown in Figure 1. Most patients were at low risk: 106 004 patients (67.6%) had a KFRE risk score at or below 1%, and 134 716 patients (86.0%) had a KFRE risk score at or below 3% (eTables 1-2 in the Supplement).

**Table 1.** Study Cohort Characteristics^a^

Characteristic	Total (N = 156 733)	KFRE	
		≤3% (n = 134 716)	>3% (n = 22 017)
Sex			
Male	64 827 (41.4)	53 824 (40.0)	11 003 (50.0)
Female	91 906 (58.6)	80 892 (60.0)	11 014 (50.0)
Race and ethnicity			
Asian	7281 (4.6)	6048 (4.5)	1233 (5.6)
Black	24 891 (15.9)	21 035 (15.6)	3856 (17.5)
Hispanic	29 658 (18.9)	24 863 (18.5)	4795 (21.8)
White	86 457 (55.2)	75 628 (56.1)	10 829 (49.2)
Unknown	8446 (5.4)	7142 (5.3)	1304 (5.9)
Index year			
2012-2013	16 477 (10.5)	13 665 (10.1)	2812 (12.8)
2014-2015	31 140 (19.9)	26 437 (19.6)	4703 (21.4)
2016-2017	47 627 (30.4)	41 023 (30.5)	6604 (30.0)
2018-2019	61 489 (39.2)	53 591 (39.8)	7898 (35.9)
CKD stage (eGFR, mL/min/1.73 m^2^)			
3a (45-59)	112 142 (71.5)	109 240 (81.1)	2902 (13.2)
3b (30-44)	37 676 (24.0)	25 137 (18.7)	12 539 (57.0)
4 (15-29)	6915 (4.4)	339 (0.3)	6576 (29.9)
Age, mean (SD), y	74.6 (8.4)	74.9 (8.1)	72.4 (10.1)
eGFR, mean (SD), mL/min/1.73 m^2^	48.8 (9.0)	51.1 (6.7)	34.9 (8.7)
ACR, median (IQR), mg/g	18.6 (58.8)	14.6 (34.3)	252.0 (914.3)

Abbreviations: ACR, albumin-creatinine ratio; CKD, chronic kidney disease; eGFR, estimated glomerular filtration rate; KFRE, Kidney Failure Risk Equation.

^a^ Data are presented as number (percentage) of patients unless otherwise indicated.

**Figure 2.**
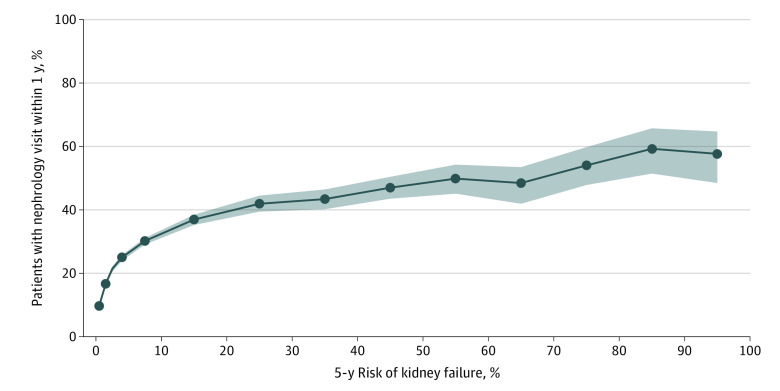
Nephrology Visit Rate, Stratified by Kidney Failure Risk Equation (KFRE) Risk We computed the nephrology visit rate as the proportion of patients who visited a nephrologist no more than 1 year after index time (date of calculable KFRE). The nephrology visit rate increases with increasing risk of 5-year kidney failure but does not exceed 65%. The shaded region represents the 95% CIs obtained from 100 bootstrapping iterations.

**Figure 3.**
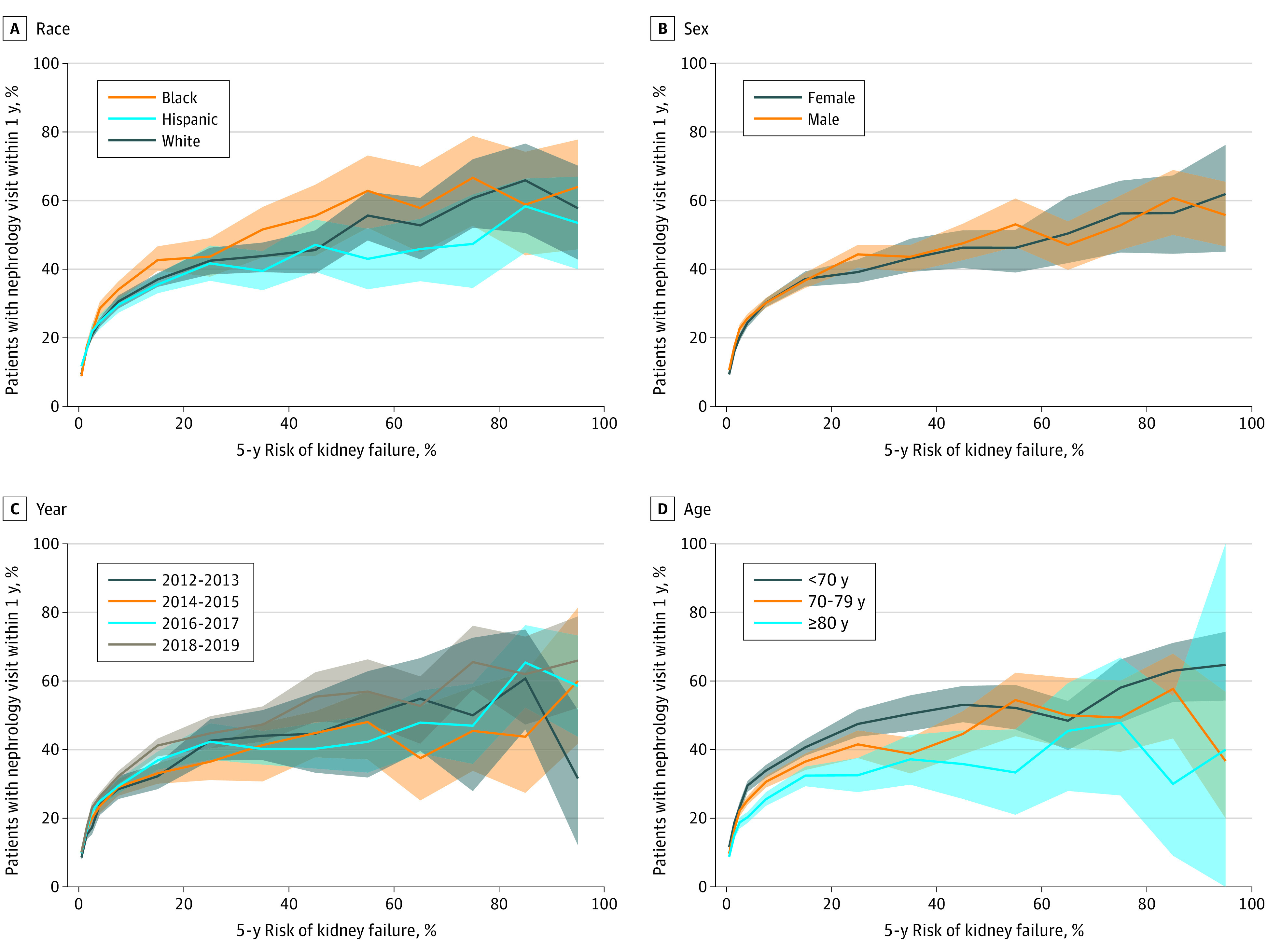
Nephrology Visit Rate, Stratified by Kidney Failure Risk Equation (KFRE) Risk and Subgroup The shaded regions represent 95% CIs obtained through 100 bootstrap iterations. The confidence bands overlap in all 4 plots, indicating that the visit rates are consistent across subgroups.

Because the KFRE model appeared reasonably well calibrated within our final patient cohort (eFigure in the Supplement), the KFRE risk score served as a credible estimate of a patient’s true risk of 5-year kidney failure. The 1-year nephrology visit rate generally increased with KFRE risk score. Across all kidney failure risk increments, the nephrology visit rate was below 65% (Figure 2). In the lowest risk group (KFRE 0%-1%), 10 409 of 106 004 patients (9.8%; 95% CI, 9.7%-10.0%) had a nephrology visit. For KFRE risk in the 20% to 30% range, 607 of 1448 patients (41.9%); 95% CI, 39.4%-44.5%) had a nephrology visit. In the highest risk group (KFRE>90%-100%), 79 of 137 patients (57.7%; 95% CI, 48.4%-64.7%) had a nephrology visit. Similar trends held across race and ethnicity, sex, index year, and age subgroups (Figure 3). Among 22 017 patients whose risk was at or above or below the 3% threshold, 7134 (32.4%; 95% CI, 31.7%-32.9%) had a nephrology visit within 1 year of index time (Table 2). At the 5% threshold, 5206 of 14 342 patients (36.3%; 95% CI, 35.4%-36.9%) had a nephrology visit, and at the 10% threshold, 3208 of 7730 patients (41.5%; 95% CI, 40.3%-42.4%) had a nephrology visit. Among 134 716 patients whose risk was at or below the 3% threshold, 15 627 (11.6%; 95% CI, 11.4%-11.7%) had a nephrology visit, and among 142 391 with risk at or below the 5% threshold, 17 514 (12.3%; 95% CI, 12.1%-12.5%) had a nephrology visit (Table 2). At or below a 10% threshold, 19 519 of 149 003 patients (13.1%; 95% CI, 12.9%-13.2%) had a nephrology visit. Patients with risk above a 3% threshold made up 14.0%, 9.2% had risk above a 5% threshold, and 4.9% had a risk above 10%.

**Table 2.** Nephrology Visit Rate According to the Kidney Failure Risk Equation Risk Thresholds^a^

Risk threshold, %	No. of patients with a nephrology visit	Total No. of patients	Nephrology visit rate, % (95% CI)
≤3	15 627	134 716	11.6 (11.4-11.7)
>3	7134	22 017	32.4 (31.7-32.9)
≤5	17 514	142 391	12.3 (12.1-12.5)
>5	5206	14 342	36.3 (35.4-36.9)
≤10	19 519	149 003	13.1 (12.9-13.2)
>10	3208	7730	41.5 (40.3-42.4)

^a^ For each risk threshold, we computed the nephrology visit rate in patients with 5-year kidney failure risk above the threshold as the proportion of patients who visited a nephrologist no more than 1 year after index time. We repeated the same analysis for patients with risk at or below the threshold. The visit rate was highest (41.5%) above the 10% risk threshold and lowest (11.6%) at or below the 3% risk threshold.

### Sensitivity Analyses

Compared with patients in our primary analysis, patients without an available ACR test result had a slightly higher mean eGFR value and lower nephrology visit rates (eTables 3-4 in the Supplement). Visit rates were higher in the analysis of prevalent nephrology care than in the primary analysis of incident nephrology care, reaching 575 of 650 patients (88.5%; 95% CI, 86.7%-91.1%) in the highest-risk group (KFRE 90%-100%). The results and interpretation of all other sensitivity analyses were not substantially different from those of the primary analysis (eTables 5-6 in the Supplement).

## Discussion

Our national study of more than 150 000 patients with CKD quantified the magnitude of the nephrology care gap for patients at risk of kidney failure. Although prior studies have pointed out low nephrology referral rates, few have provided a quantitative estimate of the gap in incident nephrology care. Our results offer insight into the opportunity for health care systems to identify and target a considerable proportion of patients with CKD who are not receiving nephrology care despite demonstrably elevated kidney risk and may help to inform future clinical and policy decisions aimed at bridging this gap.

The 1-year nephrology visit rate generally increased with KFRE risk score, but the absolute rates were low. The visit rate among patients in the highest-risk groups (5-year KFRE 80%-100%) was only approximately 60%, implying that there may be inadequate delivery of nephrologist care to even the most high-risk patients. Furthermore, only modest increases in visit rate occurred above 20% KFRE risk: although the proportion with a nephrology visit was 41.9%in the 20% to 30% risk category, it increased to only approximately 60% in the highest-risk groups. This finding suggests that referring clinicians may not distinguish varying risk strata within high-risk patients with CKD. Risk stratification using the KFRE could provide added information about likelihood of progression and help clinicians make referral decisions that are tailored more precisely to a patient’s needs.

Although wide consensus is lacking on the kidney failure risk threshold at which patients should be referred to a nephrologist, a 5-year KFRE threshold of 3% was used in Manitoba, Canada for triage of referrals to a nephrologist, and the UK the National Institute for Health and Care Excellence guidelines recently added a 5% threshold to recommended indications for nephrology referral.^10,15,18^ In our study, above thresholds of 3% and 5%, less than half of patients had visited a nephrologist within 1 year. Even above a threshold of 10%, only 41.5% of patients had a nephrologist visit. The fact that 4.9% of patients had a KFRE risk that exceeded 10% means that 2 of every 100 patients with CKD had a very high risk of kidney failure yet did not receive nephrology care within 1 year of established risk. If a threshold for referral were instituted, we hypothesize that a sizable number of patients would receive nephrology care who would not have otherwise, many of whom could have preventable CKD progression. We also observed that 11.6% to 13.1% of patients with risk at or below the 3%, 5%, and 10% risk thresholds saw a nephrologist. The majority of patients (86.0%) had a KFRE risk less than 3%, which means that every 10 of 100 patients with CKD had low risk of kidney failure yet still visited a nephrologist. Although some visits may have been warranted for reasons unrelated to kidney failure risk, it is possible that some low-risk patients received nephrology care that did not add value to their treatment. Risk stratification with KFRE could reduce unnecessary low-risk referrals. Even decreasing the low-risk referral rate by 10% to 20% would improve capacity for nephrology services to accommodate the 2 of 100 higher-risk patients who need to be seen.

Several factors may contribute to the gap in nephrology care. First, underreferral may play a role, and use of a KFRE risk threshold concurrently with other important laboratory-based indicators could help primary care clinicians identify and refer patients whose high risk for CKD progression may not be apparent from traditional criteria alone (eg, eGFR). Another possible cause of the observed gap in care could be a lack of follow-through in patients who had a referral placed. Improvement of referral follow-through can be accomplished by reducing wait times for a nephrology visit and expanding access to underserved areas with geographic barriers.^23^ A known shortage in nephrologists may have also contributed to limited access to nephrologist care.^24,25^ High demand relative to limited nephrologist availability may be addressed by leveraging automated clinical decision support tools that combine KFRE risk stratification with diagnostic and therapeutic steps for low-risk patients who may be safely managed in the primary care setting without nephrology referral. For these patients, the main complication of CKD is elevated risk of CVD, and management is focused on CVD risk assessment, medical therapy to reduce CVD risk (eg, statins and blood pressure control), and lifestyle interventions (eg, weight loss, dietary counseling, and smoking cessation).^26^ With management of low-risk CKD contained in the primary care setting, nephrology resources could be better concentrated toward caring for patients with CKD at high risk of kidney failure.

Urine ACR is a necessary input to calculating KFRE risk and is informative for identifying and treating patients at risk for developing CVD. However, there is a well-documented underuse of ACR testing, which was also observed in our own study.^16,27^ Electronic health record–embedded decision support systems that harness information on family history and preexisting conditions could alert primary care clinicians as to when ACR testing should be prioritized. Risk evaluation for CVD and KFRE could then take place as second-line triages after ACR testing has been recommended.

Of interest, the gap in nephrology care was more pronounced in incident than in prevalent nephrology care, which suggests that patients at high risk who have not previously been seen by a nephrologist are systematically more likely to continue to not be seen. Future work could examine whether embedded risk prediction and automatic referrals might help bring appropriate nephrology care to previously unreferred and unseen high-risk patients.

### Limitations

The claims-based data set did not allow us to disentangle the extent to which the low visit rates were driven by underreferral, loss to follow-up, or insufficient nephrologist availability to meet demand. Our estimate of the gap in care may also be biased if consultations are occurring through mechanisms that are not reliably captured in the claims database, such as electronic consultations. In addition, we included only patients with an eGFR less than 60 mL/min/1.73 m^2^ because this is the population in which the KFRE has been most extensively validated. We did not include patients with an eGFR of 60 mL/min/1.73 m^2^ or higher who may have had extreme albuminuria or other indications that still warrant nephrology care. A further limitation was that only 25% of patients with an eGFR less than 60 mL/min/1.73 m^2^ had a urine ACR within 90 days. We mitigated the effects of missing ACR in a sensitivity analysis by including patients with a urine protein-creatinine ratio measurement (converted to urine ACR). Furthermore, because nephrology visit rates were even lower in patients without an ACR than in patients with an ACR, we suspect including these patients would further emphasize the gap in nephrology care. Finally, our patient cohort consisted of individuals who had Medicare Advantage or commercial insurance, and the nephrology visit rates from our analysis may not be applicable to noncommercially insured or uninsured populations.

## Conclusions

The findings of this cohort study indicate that in the US, a gap exists in the delivery of nephrologist care to patients with CKD: among patients who are at high risk of kidney failure, nearly half do not visit a nephrologist within a year of being established as high-risk patients. This gap in care demonstrates a clear need for strategies that appropriately identify patients who could benefit from referral to nephrology care and to deliver targeted intervention to these patients. Accomplishing such an improvement in care delivery may require integration of risk assessment and triage tools into electronic health records, programs that deter loss of follow-through, and use of digital decision support technology to concentrate nephrology services for the highest-risk patients.

## Notes

Supplement. **eMethods.** Supplementary Methods **eFigure.** KFRE Calibration **eTable 1.** Distribution of KFRE Risk Scores **eTable 2.** Subgroup Composition of KFRE Risk Groups **eTable 3.** Characteristics of Patients in Primary Analysis Population Versus Patients With Missing ACR **eTable 4.** Nephrology Visit Rates Across CKD Stages in Primary Analysis Population Versus Patients With Missing ACR **eTable 5.** Nephrology Visit Rates in Groups Above and Below 3%, 5%, and 10% Risk Thresholds, Sensitivity Analysis **eTable 6.** Nephrology Visit Rates Across KFRE Risk Groups, Sensitivity Analysis
eReferences
