# Supplementary material for: Analysis of Specialty Nephrology Care Among Patients With Chronic Kidney Disease and High Risk of Disease Progression
Source: JAMA Netw Open. 2022 Aug 19;5(8):e2225797. doi: 10.1001/jamanetworkopen.2022.25797 (PMC9391959; doi:10.1001/jamanetworkopen.2022.25797)
Supplement: Supplement. — eMethods. Supplementary Methods eFigure. KFRE Calibration eTable 1. Distribution of KFRE Risk Scores eTable 2. Subgroup Composition of KFRE Risk Groups eTable 3. Characteristics of Patients in Primary Analysis Population Versus Patients With Missing ACR eTable 4. Nephrology Visit Rates Across CKD Stages in Primary Analysis Population Versus Patients With Missing ACR eTable 5. Nephrology Visit Rates in Groups Above and Below 3%, 5%, and 10% Risk Thresholds, Sensitivity Analysis eTable 6. Nephrology Visit Rates Across KFRE Risk Groups, Sensitivity Analysis eReferences [file jamanetwopen-e2225797-s001.pdf]

Supplementary Online Content

Wang M, Peter SS, Chu CD, Tuot DS, Chen JH. Analysis of specialty nephrology care among patients with chronic kidney disease and high risk of disease progression. *JAMA Netw Open*. 2022;5(8):e2225797. doi:10.1001/jamanetworkopen.2022.25797

eMethods. Supplementary Methods
eFigure. KFRE Calibration
eTable 1. Distribution of KFRE Risk Scores
eTable 2. Subgroup Composition of KFRE Risk Groups
eTable 3. Characteristics of Patients in Primary Analysis Population Versus Patients With Missing ACR
eTable 4. Nephrology Visit Rates Across CKD Stages in Primary Analysis Population Versus Patients With Missing ACR
eTable 5. Nephrology Visit Rates in Groups Above and Below 3%, 5%, and 10% Risk Thresholds, Sensitivity Analysis
eTable 6. Nephrology Visit Rates Across KFRE Risk Groups, Sensitivity Analysis
eReferences

This supplementary material has been provided by the authors to give readers additional information about their work.

© 2022 Wang M et al. *JAMA Network Open.*

eMethods. Supplementary Methods

Sensitivity Analyses

Excluding patients with a nephrology visit in the last three years: It is possible that excluding patients with a nephrology visit within the past year may not be a long enough timeframe to accurately measure the rates of incident nephrology care. Some patients may have had appropriate reasons not to see a nephrologist within one year of a previous visit. We thus extended the timeframe of exclusion from one year to three years and calculated the visit rates for this smaller patient population.

Prevalent nephrology care: In our primary analysis, we excluded patients with a nephrology visit within one year prior to index time and calculated visit rates using visits to nephrology *after* index time. This approach captured the rates of *incident* nephrology care, i.e. care administered to patients who had not been previously referred or seen. To evaluate the rates of *prevalent* nephrology care, we kept patients with visits to nephrology within one year prior to index time and calculated a “two-sided” nephrology visit rate as the fraction of patients with a visit to nephrology within one year prior *or* one year after index time.

Kaplan-Meier analysis: Because the outcome of our primary was defined by having a nephrology visit within one year of the index date, we only included patients who had at least one year of follow-up time. However, this may bias the results if excluded patients (those who disenrolled or died within one year) differ substantially from retained patients. To examine this possible bias, we reintroduced the patients that had been excluded due to death or disenrollment within one year and used Kaplan-Meier survival models to determine the one-year cumulative incidence of nephrology visits.

Urine PCR to urine ACR conversion: Many patients were excluded from the primary analysis due to not having an available ACR measurement. To address this limitation and construct a larger, more representative cohort, we utilized a validated conversion equation to convert urine PCR measurements to urine ACR values.[1]

Visit rates in patients without an available ACR: If patients without an available ACR measurement differed systematically from patients with available an ACR measurement, then excluding the former could bias our estimates of visit rates. To address this concern, we calculated visit rates using the date of eGFR result as index time for (1) patients with eGFR < 60 mL/min/1.73 m^2 and (2) patients with eGFR < 60 mL/min/1.73 m^2 and missing ACR. With (1), we also addressed the concern that requiring a calculable KFRE may skew index time: that is, patients may have been referred for nephrology care using an eGFR value alone, and ACR measurements only became available after the nephrology visit.

2009 CKD-EPI: KFRE was originally developed to use eGFR calculated from the 2009 version of the CKD-EPI equation, rather than the 2021 version.[2] However, the 2009 version is now discouraged due to its use of race as an input variable.[3] To determine whether the version of the CKD-EPI equation affected the results, we calculated nephrology visit rates across KFRE scores that used eGFR values computed with the 2009 CKD-EPI equation.

© 2022 Wang M et al. *JAMA Network Open.*

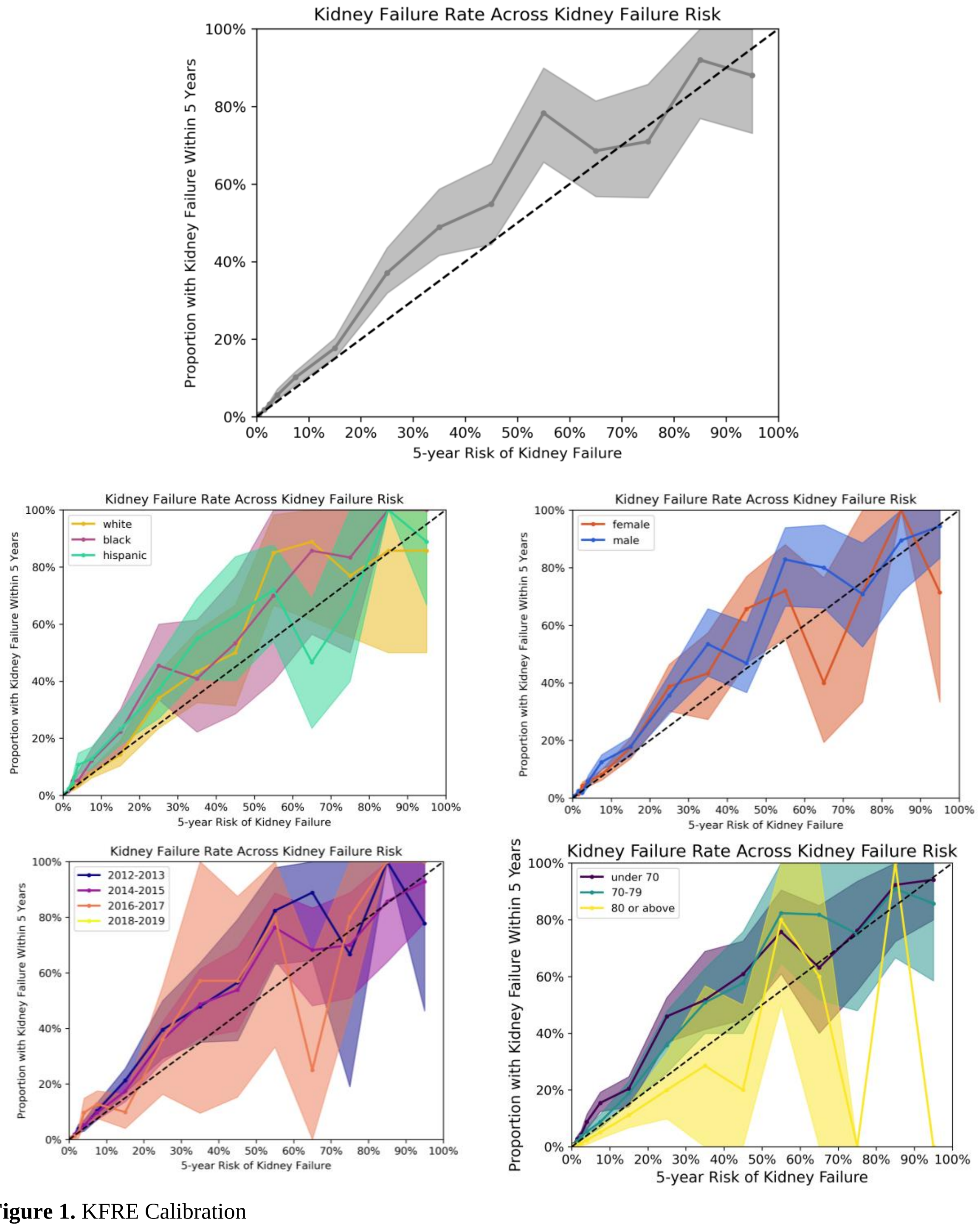

eFigure 1. KFRE Calibration

We assessed the calibration of the KFRE risk score by comparing the true rate of 5-year kidney failure to the KFRE estimated risk of kidney failure. Within each risk group, we computed the probability of kidney failure occurring within 5 years as the number of individuals in the group who underwent dialysis or transplant within 5 years of index time divided by the total number of individuals in the group. A well-calibrated score should fall close to the y=x line (dashed line in plots). We observed that the KFRE risk score was reasonably well-calibrated, both overall and within subgroups. The “2018-2019” subgroup was omitted due to unobserved 5-year follow up, as data were only available through 2021. Similarly, the “80 or above” subgroup had high variability due to small patient numbers.

© 2022 Wang M et al. *JAMA Network Open.*

Risk Group	Patients in Group	
	N (95% CI)	% of Total (95% CI)
< 1%	106004 (105649, 106362)	67.6 (67.4, 67.9)
> 1%, ≤ 2%	20272 (20010, 20467)	12.9 (67.4, 67.9)
> 2%, ≤ 3%	8440 (8271, 8600)	5.38 (5.28, 5.49)
> 3%, ≤ 5%	7675 (7568, 7880)	4.90 (4.83, 5.03)
> 5%, ≤ 10%	6612 (6406, 6769)	4.22 (4.09, 4.32)
> 10%, ≤ 20%	3819 (3716, 3955)	2.44 (2.37, 2.52)
> 20%, ≤ 30%	1448 (1385, 1521)	0.92 (0.88, 0.97)
> 30%, ≤ 40%	795 (738, 851)	0.51 (0.47, 0.54)
> 40%, ≤ 50%	515 (472, 573)	0.33 (0.30, 0.37)
> 50%, ≤ 60%	339 (303, 373)	0.22 (0.19, 0.24)
> 60%, ≤ 70%	291 (256, 333)	0.19 (0.16, 0.21)
> 70%, ≤ 80%	224 (200, 248)	0.14 (0.13, 0.16)
> 80%, ≤ 90%	162 (138, 185)	0.10 (0.09, 0.12)
> 90%, ≤ 100%	137 (117, 161)	0.09 (0.07, 0.10)

eTable 1. Distribution of KFRE Risk Scores
We subdivided the patient cohort into risk increments of 10%. Because the 0 to 10% risk range contains 95% of the patients, we further subdivided these patients into finer-grained increments of 0 to 1%, >1 to 2%, >2 to 3%, >3 to 5%, and >5 to 10%. Most patients had risk in the 0 to 1% range. The 95% confidence interval was obtained from 100 bootstrapping iterations.

© 2022 Wang M et al. *JAMA Network Open.*

Risk Group	Race/Ethnicity (%)			Sex (%)		Age (%)			Index year (%)			
	White	Black	Hispanic	Male	Female	< 70	70-79	≥ 80	’12-’13	’14-’15	’16-’17	‘17-’18
< 1%	56.6	15.5	18.4	38.3	61.7	23.0	44.9	32.1	9.8	19.3	30.5	40.4
> 1%, ≤ 2%	55.2	16.0	18.4	45.8	54.2	26.7	41.2	32.1	11.4	20.6	30.2	37.7
> 2%, ≤ 3%	53.1	16.7	19.2	47.2	52.8	28.2	40.9	30.9	11.6	21.3	30.1	37.0
> 3%, ≤ 5%	53.0	17.2	19.5	47.3	52.7	31.0	38.2	30.8	12.5	21.0	30.3	36.2
> 5%, ≤ 10%	49.9	16.9	21.3	49.7	50.3	33.0	37.8	29.2	12.4	21.4	29.9	36.3
> 10%, ≤ 20%	48.5	17.1	22.8	50.2	49.8	35.9	38.3	25.8	13.3	21.6	29.7	35.3
> 20%, ≤ 30%	44.5	19.1	23.7	53.5	46.5	41.0	36.3	22.7	14.4	22.0	28.7	34.9
> 30%, ≤ 40%	43.9	19.7	25.2	52.2	47.8	42.1	38.2	19.6	13.7	21.0	30.7	34.6
> 40%, ≤ 50%	41.7	19.2	26.8	55.5	44.5	47.6	34.0	18.4	12.6	22.5	30.9	34.0
> 50%, ≤ 60%	36.6	20.6	27.4	52.8	47.2	54.3	29.8	15.9	12.4	22.7	28.6	36.3
> 60%, ≤ 70%	37.1	22.0	29.2	58.4	41.6	54.0	30.9	15.1	10.7	19.2	32.3	37.8
> 70%, ≤ 80%	37.5	18.8	25.4	64.3	35.7	55.4	34.4	10.3	7.1	24.6	29.5	38.8
> 80%, ≤ 90%	27.2	21.0	37.0	66.0	34.0	61.7	32.1	6.2	17.3	19.8	32.1	30.9
> 90%, ≤ 100%	32.8	18.2	31.4	69.3	30.7	74.5	21.9	3.6	13.9	21.9	29.9	34.3

eTable 2. Subgroup Composition of KFRE Risk Groups
For each risk group, we determined the percentage breakdown in race/ethnicity, sex, age, and index year.

© 2022 Wang M et al. *JAMA Network Open.*

	Two eGFR < 60	*(Primary Analysis)* **Two eGFR < 60 and Available ACR**	**Two eGFR < 60 and Missing ACR**
Total	(n=632,767)	(n=156,733)	(n=476,034)
Sex	N (%)		
Male	240,375 (38.0)	64,827 (41.4)	175548 (36.9)
Female	392,392 (62.0)	91,906 (58.6)	300486 (63.1)
Race/Ethnicity	N (%)		
White	404,423 (63.9)	86,457 (55.2)	317,966 (66.8)
Black	101,858 (16.1)	24,891 (15.9)	76,967 (16.2)
Hispanic	74,326 (11.7)	29,658 (18.9)	44,668 (9.4)
Asian	19,686 (3.1)	7,281 (4.6)	12,405 (2.6)
Unknown	32,474 (5.1)	8,446 (5.4)	24,028 (5.0)
Age	Mean (SD)		
	74.4 (9.1)	74.6 (8.4)	74.4 (9.3)
eGFR (mL/min/1.73 m^2)	Mean (SD)		
	49.9 (8.57)	48.8 (9.0)	50.3 (8.4)
CKD Stage	N (%)		
Stage 3a (eGFR = 45-59 mL/min/1.73 m^2)	481,534 (76.1)	112,142 (71.5)	369,392 (77.6)
Stage 3b (eGFR = 30-44 mL/min/1.73 m^2)	129,303 (20.4)	37,676 (24.0)	91,627 (19.2)
Stage 4 (eGFR = 15-29 mL/min/1.73 m^2)	21,930 (3.5)	6,915 (4.4)	15,015 (3.2)

eTable 3. Characteristics of Patients in Primary Analysis Population Versus Patients With Missing ACR
In our primary analysis, we examined only patients with a calculable KFRE, defined as those with two eGFR values less than 60 mL/min/1.73m^2 and an ACR measurement within ± 90 days of the eGFR result. To assess whether this inclusion criteria may have biased our results, we compared the characteristics of our primary analysis population to (1) all patients with two eGFR < 60 mL/min/1.73m^2 and (2) patients who did not have an ACR measurement within ± 90 days of an eGFR result.

© 2022 Wang M et al. *JAMA Network Open.*

	Two eGFR < 60	*(Primary Analysis)* **Two eGFR < 60 and Available ACR**	**Two eGFR < 60 and Missing ACR**
CKD Stage	Nephrology Visit Rate, %		
Stage 3a (eGFR = 45-59 mL/min/1.73 m^2)	19.4	25.4	17.5
Stage 3b (eGFR = 30-44 mL/min/1.73 m^2)	35.5	41.6	33.0
Stage 4 (eGFR = 15-29 mL/min/1.73 m^2)	53.4	56.1	52.1

eTable 4. Nephrology Visit Rates Across CKD Stages in Primary Analysis Population Versus Patients With Missing ACR

We calculated the nephrology visit rate across CKD stages in (1) all patients with two eGFR < 60 mL/min/1.73m^2 and (2) patients who did not have an ACR measurement within ± 90 days of an eGFR result. Notably, compared to our primary analysis population, nephrology visit rates in these patient groups were lower.

© 2022 Wang M et al. *JAMA Network Open.*

Risk Category	Nephrology Visit Rate (95% CI)				Cumulative Nephrology Visit Incidence (95% CI)
	Exclude 3 Years (n=143,398)	Prevalent (n=231,382)	Include UPCR (n=174,335)	2009 CKD-EPI (n=186,199)	Kaplan-Meier (n=176,742)
<3%	10.6 (10.4, 10.7)	36.3 (36.0, 36.5)	14.1 (13.9, 14.3)	10.5 (10.3, 10.6)	11.9 (11.7, 12.1)
≥3%	30.8 (30.1, 31.3)	70.8 (70.4, 71.2)	37.5 (36.9, 37.9)	30.5 (29.9, 31.0)	33.7 (33.1, 34.3)
<5%	11.2 (11.1, 11.4)	38.3 (38.0, 38.5)	15.0 (14.9, 15.2)	11.2 (11.0, 11.3)	12.7 (12.5, 12.9)
≥5%	34.7 (33.7, 35.4)	74.4 (74.0, 74.9)	41.2 (40.4, 41.8)	34.4 (33.6, 35.0)	37.7 (37.0, 38.4)
<10%	12.0 (11.8, 12.1)	40.3 (40.1, 40.5)	16.1 (15.9, 16.3)	11.9 (11.8, 12.1)	13.6 (13.4, 13.7)
≥10%	40.1 (38.9, 41.1)	78.5 (77.9, 79.1)	45.8 (44.8, 46.6)	39.5 (38.3, 40.5)	43.3 (42.2, 44.3)

eTable 5. Nephrology Visit Rates in Groups Above and Below 3%, 5%, and 10% Risk Thresholds, Sensitivity Analysis

For our sensitivity analysis, we computed the nephrology visit rates above referral thresholds in populations derived with criteria that were modified from those used to construct the primary analysis population. See Supplemental Methods for details on the alterations made to the criteria. We note that only the prevalent nephrology care sensitivity analysis yielded different visit rates, which we comment on in Discussion. The 95% confidence interval was obtained from 100 bootstrapping iterations.

© 2022 Wang M et al. *JAMA Network Open.*

Risk Group	Nephrology Visit Rate (95% CI)				Cumulative Nephrology Visit Incidence (95% CI)
	Exclude 3 Years (n=143,398)	Prevalent (n=231,382)	Include UPCR (n=174,335)	2009 CKD-EPI (n=186,199)	Kaplan-Meier (n=176,742)
< 1%	8.9 (8.7, 9.0)	30.9 (30.6, 31.2)	11.6 (11.4, 11.8)	8.8 (8.7, 8.9)	10.1 (9.9, 10.3)
> 1%, ≤ 2%	15.7 (15.2, 16.3)	49.1 (48.5, 49.5)	20.9 (20.5, 21.4)	15.5 (15.1, 16.0)	17.1 (16.6, 17.5)
> 2%, ≤ 3%	20.1 (19.3, 21.2)	56.7 (55.9, 57.5)	26.4 (25.5, 27.2)	19.4 (18.6, 20.2)	21.9 (21.2, 22.8)
> 3%, ≤ 5%	24.0 (22.9, 24.9)	61.9 (61.0, 62.5)	29.7 (28.8, 30.4)	23.5 (22.5, 24.4)	25.9 (25.0, 26.6)
> 5%, ≤ 10%	28.5 (27.2, 29.6)	68.1 (67.4, 69.0)	35.4 (34.2, 36.2)	28.5 (27.7, 29.6)	31.1 (30.1, 32.1)
> 10%, ≤ 20%	35.8 (34.1, 37.4)	74.3 (73.3, 75.0)	41.7 (40.2, 43.0)	34.6 (32.9, 35.8)	38.4 (36.7, 39.5)
> 20%, ≤ 30%	40.4 (37.3, 43.1)	77.7 (76.4, 78.9)	45.6 (43.5, 47.5)	38.7 (36.4, 40.6)	43.9 (42.0, 46.8)
> 30%, ≤ 40%	41.1 (37.1, 44.3)	80.8 (79.1, 82.5)	47.8 (45.5, 50.2)	44.6 (41.7, 47.5)	45.9 (43.2, 49.5)
> 40%, ≤ 50%	47.9 (43.4, 52.3)	82.6 (80.8, 84.7)	49.0 (45.2, 52.3)	44.7 (41.0, 48.4)	48.1 (44.6, 51.9)
> 50%, ≤ 60%	47.5 (41.7, 53.7)	83.9 (81.8, 85.9)	52.3 (48.1, 55.7)	48.2 (43.4, 52.4)	50.8 (46.0, 55.6)
> 60%, ≤ 70%	45.6 (38.7, 52.8)	84.6 (82.4, 86.8)	53.7 (49.3, 58.0)	52.6 (47.2, 57.6)	50.6 (45.7, 54.1)
> 70%, ≤ 80%	52.3 (45.6, 58.7)	86.6 (84.1, 88.7)	58.3 (53.2, 62.8)	48.2 (43.2, 54.1)	57.4 (51.9, 63.9)
> 80%, ≤ 90%	59.9 (50.7, 66.5)	86.1 (83.6, 88.6)	53.5 (47.9, 59.9)	54.8 (48.0, 60.4)	64.7 (58.3, 71.9)
> 90%, ≤ 100%	56.0 (47.9, 65.8)	88.5 (86.7, 91.1)	62.4 (56.0, 70.0)	59.5 (52.7, 66.4)	62.2 (52.1, 70.3)

eTable 6. Nephrology Visit Rates Across KFRE Risk Groups, Sensitivity Analysis
For our sensitivity analysis, we computed the nephrology visit rates in populations derived with criteria that were modified from those used to construct the primary analysis population. See Supplemental Methods for details on the alterations made to the criteria. We note that only the prevalent nephrology care sensitivity analysis yielded different visit rates, which we comment on in Discussion. The 95% confidence interval was obtained from 100 bootstrapping iterations.

© 2022 Wang M et al. *JAMA Network Open.*

eReferences

1. Sumida K, Nadkarni GN, Grams ME, et al. Conversion of Urine Protein-Creatinine Ratio or Urine Dipstick Protein to Urine Albumin-Creatinine Ratio for Use in Chronic Kidney Disease Screening and Prognosis : An Individual Participant-Based Meta-analysis. *Ann Intern Med*. 2020;173(6):426-435.
2. Levey AS, Stevens LA, Schmid CH, et al. A new equation to estimate glomerular filtration rate. Ann Intern Med. 2009;150(9):604-612.
3. Inker LA, Eneanya ND, Coresh J, et al. New Creatinine- and Cystatin C-Based Equations to Estimate GFR without Race. N Engl J Med. Published online September 23, 2021. doi:10.1056/NEJMoa2102953.

© 2022 Wang M et al. *JAMA Network Open.*
